# Entropy Decoding the Fundamental Law of Phase Competition in Glass Formation

**DOI:** 10.1002/advs.75936

**Published:** 2026-06-15

**Authors:** Benke Huo, Zhengqing Cai, Bingtao Wang, Zhenqiang Song, Shi‐Dong Feng, Zijing Li, Xingjun Liu, Li‐Min Wang

**Affiliations:** ^1^ Center for Advanced Structural Materials State Key Laboratory of Metastable Materials Science and Technology Yanshan University Qinhuangdao P. R. China; ^2^ Key Laboratory for Microstructural Material Physics of Hebei Province School of Science Yanshan University Qinhuangdao Hebei P. R. China; ^3^ School of Materials Science and Engineering Harbin Institute of Technology Shenzhen P. R. China; ^4^ School of Materials Science and Engineering Hebei University of Technology Tianjin P. R. China

**Keywords:** entropy engineering, glass formation, melting entropy, metallic glass, phase competition

## Abstract

The quest to understand the fundamentals of optimal glass‐forming compositions in multi‐component alloys has long been challenging. To gain insights into the mechanism, a systematic study of glass compositions is conducted using a new strategy of entropy engineering, imposed by the integration of eutectic or intermetallic phases featured by their low melting entropies, which can be determined experimentally and precisely. The optimal composition designed using entropy engineering is further compared with that derived from the conventional “deep eutectic” principle and empirical trial‐and‐error. For the ternary Cu‐Zr‐Ti alloys, a series of compositions are achieved, and the ranking of their glass‐forming ability indicates a correlation with the melting entropies of initial phases. In particular, the optimal glass‐forming composition of Cu_59.99_Zr_28.75_Ti_11.26_ is designed using two initial phases of intermetallic Cu_50_Zr_50_ and eutectic Cu_73.5_Ti_26.5_ with the lowest melting entropies. This designed composition is remarkably equivalent to the reported one, Cu_60_Zr_30_Ti_10_. It is more meaningful that this study uncovers the phase competition mechanism involved in glass formation in a quantitative way for the first time, emphasizing the importance of melting entropies in screening and balancing the competing phases upon glass formation.

## Graphical abstract

We validate the integration of intermetallic and eutectic phases as initial phases for composition design. The phase competition mechanism in glass formation is quantitatively clarified based on the melting entropy of competing phases. Glass‐forming ability is modulated by tuning phase competition via the melting entropy of initial phases. A highly efficient entropy‐engineering framework is established for predicting and rationally designing metallic glass compositions.

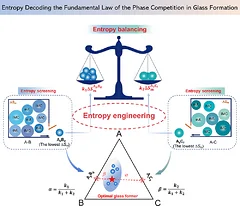

## 1 Introduction

The clarification of the fundamental laws of the optimal glass‐forming compositions in multicomponent alloys has been a pivotal challenge in the field of metallic glasses (MGs) [1, 2, 3, 4, 5, 6, 7, 8]. Traditionally, compositions adjacent to the eutectic points of phase diagrams have been regarded as ideal candidates for glass formation due to their suppressed crystallization tendency and low liquidus temperatures [9, 10]. This perspective arises from empirical understanding that the eutectic compositions generally have higher degree of supercooling, lower thermodynamic driving forces for crystallization, and higher melting viscosity, all of which are conducive to glass formation [11, 12]. However, in the multiple‐eutectic phase diagram, it is occasionally puzzling to determine which eutectic points should be chosen for the design of MGs, since, experimentally, the glass‐forming ability (GFA) of the eutectic composition with the lowest eutectic temperature might not necessarily be the optimal glass former [13]. Moreover, an increasing number of evidences challenge the conventional “deep eutectic” paradigm, as the optimal glass‐forming compositions frequently exhibit off‐eutectic behaviors [3, 14]. For instance, in binary Cu‐Zr alloys, the highest GFA occurs at off‐eutectic compositions, most notably at Cu_50_Zr_50_, which corresponds to an intermetallic compound rather than a eutectic composition [15, 16]. This apparent contradiction highlights the unresolved nature of the fundamental laws governing optimal glass‐forming. Over decades, numerous empirical criteria have been proposed to rationalize glass formation, including atomic size mismatch [17], negative mixing enthalpy [18], and topological instability [19]. However, none of these descriptors fully explain the mechanisms underlying both eutectic and off‐eutectic glass formation. However, among commonly used parameters such as low melting temperature (*T*_m_), high liquidus viscosity (*η*_Tl_), and high reduced glass transition temperature (*T*_rg_), only a partial correlation with eutectic compositions has been observed [20, 21, 22, 23, 24, 25, 26, 27, 28, 29], but these parameters do not work well consistently in evaluating glass formation and predicting the optimal composition [30, 31, 32, 33, 34].

The lack of a quantitative framework capable of accurately describing optimal glass‐forming behaviors remains a major limitation in the development of MGs. Notably, the viewpoint of competition among multiple crystalline phases during solidification may shed light on the essence of the “deep eutectic” principles and off‐eutectic behaviors in the multi‐component alloys [35, 36]. It is argued that multiple crystalline phases would be involved during crystallization, which requires to overcome greater energy barriers and long‐range atomic migration due to the different atomic types and ratios, playing a crucial role for the diverse precipitations upon solidification [37].

Experimental and computational studies support this view that specific competition of crystalline phases would help the metallic melts bypass crystallization and eventually reach to glassy phases [38, 39, 40]. For instance, in the Zr‐Cu‐Al alloys, when the crystalline phases of ZrCu and ZrAl become dominant competition phases, GFA can be significantly enhanced [41]. It has also been mentioned that phase competition will be further intensified by increasing the number of elements and atomic size differences, and then the confused orders lead to frustration of crystallization [42, 43]. The idea of phase competition helps greatly advances the understanding of the glass formation mechanism for MGs.

Despite its conceptual significance, the notion of phase competition upon glass formation remains largely qualitative, which makes it difficult for the development of MGs to break free from the time‐consuming situation of trial‐and‐error research. There are even extreme cases where vitrification can be hardly achieved through enhancing phase competition by microalloying dozens of elements within the systems [44]. Reasoning this dilemma concerns the basic laws of the competitive crystalline phases conducive to glass formation, and the manipulation and design of the competition of crystalline phases so as to maximize the competitive effect for glass formation.

In this study, we seek to quantify the phase competition mechanism from the perspective of melting entropy (Δ*S*_m_) of crystalline phases. We propose that Δ*S*_m_ can serve as an effective indicator and weight coefficient for evaluating the relative stability of competing crystalline phases during solidification. This approach stems from our finding that Δ*S*_m_ of crystalline phases correlates closely with glass formation and provides a holistic descriptor for glass formation [45]. To validate this concept, we conduct a comprehensive investigation on ternary Cu‐Zr‐Ti alloys, analyzing their glass‐forming compositions and phase competition behaviors. The results demonstrate that entropy engineering, the tuning of glass formation through control of melting entropies, offers a reliable and quantitative pathway for designing MGs based on phase competition.

## 2 Entropy Engineering in Composition Design

Moving beyond traditional methods for MG development, we introduce an “entropy engineering” approach centered on the melting entropy (Δ*S*_m_). This parameter, characterizing the entropy change during solid–liquid transition, In the theoretical framework of entropy engineering, the parameter of Δ*S*_m_ is taken as the primary criterion for selecting initial phases and to balance the weighting among the competitive phases for designing MGs. Our recent studies have shown that Δ*S*_m_ throws fundamental effect on glass formation, as reflected by its accordance with other key factors affecting the solidification process. For example, when ranking the GFA among molecular isomers, it is found that Δ*S*_m_ relates more closely to GFA than melting point, *T*_m_, which has been specially focused in the traditional design of MGs [45]. According to Turnbull's approximation, Δ*G* = Δ*S*_m_ (*T*_m—_ *T*), a lower value of Δ*S*_m_ appears to correlate to a reduced driving force (Δ*G*) for crystallization [28]. Given that the fast crystallization temperature zone in supercooled liquids is generally located at 0.79 *T*_m_ [46], it is natural that Δ*S*_m_ is crucial to determine Δ*G*. In particular, our recent study also reveals that a low‐Δ*S*_m_ is intrinsically linked to higher kinetic viscosity of the liquid phase, resulting in sluggish atomic kinetics that favor glass formation [47]. Considering the relations between the Δ*S*_m_ and other key factors influencing glass formation, a low‐Δ*S*_m_ emerges as a defining thermodynamic characteristic of crystalline alloys prone to glass formation, establishing Δ*S*_m_ as the key descriptor in the present entropy‐engineering framework.

Based on this principle, the initial phases for alloy design are selected from intermetallic compounds (IMC) or eutectic (eut.) compositions with low‐Δ*S*_m_. The preference for eutectic compositions over off‑eutectic ones in this selection is justified by their synergistic thermodynamic and kinetic advantages. As evidenced in the Cu‐Ag system, where peak GFA at the eutectic composition correlates with enhanced local structural order and retarded atomic mobility [48], eutectic melts generally combine intrinsically lower Δ*S*_m_ and reduced liquidus temperatures, which not only diminish the crystallization driving force but also coincide with densely packed local structures (e.g., five‑fold symmetry) and more sluggish melt dynamics. This approach overcomes the limitations of traditional methods that rely predominantly on metallic element interactions. The balance between these chosen phases is determined by their respective Δ*S*_m_, i.e., the mole ratio of the selected initial phases was leveraged by Δ*S*_m_, as follows,

(1) $$\alpha \Delta S_{m\alpha }=\beta \Delta S_{m\beta }$$

where *α* and *β* represent the proportions of the selected initial phases A and B, respectively, the sum of *α* and *β* equals 1. Thereby, the nominal composition of the designed alloy can be calculated from the values of *α* and *β* and the compositions of the phases A and B. The entropy engineering is completely different from the traditional design methods of MGs. First, this approach involves designing MGs from binary phases, rather than traditionally by using pure elements, which greatly enhances the designing efficiency of MGs. In particular, only binary phases possessing high crystallization barriers are employed, since the precipitation of pure elements or simple solid solutions generally lacks the kinetic resistance required to effectively impede crystallization. By preselecting phases intrinsically resistant to crystallization, this strategy substantially enhances the ability to stabilize the amorphous state. Furthermore, this approach advances the design of MGs from enthalpy‐controlled schemes merely focusing on the interatomic interactions to the entropy‐controlled scheme that governs crystallization. This represents a fundamental shift from the traditional, enthalpy‐dominant philosophy, which emphasizes metallic element interactions.

The implementation of this entropy‐weighted framework requires a reliable determination of Δ*S*_m_ for the selected binary phases. Although the CALPHAD method can in principle, estimate multicomponent melting entropies, its accuracy is limited by systematic uncertainties arising from the extrapolation of binary Gibbs energy descriptions into multicomponent systems. As shown in Table S1, noticeable deviations exist between CALPHAD predictions and experimental values for key competing phases such as Cu_10_Zr_7_ and Cu_10_Hf_7_. To ensure thermodynamic consistency in the entropy‐balancing process, experimentally measured thermodynamic parameters are therefore adopted as the primary inputs in the present framework, while CALPHAD is retained as an auxiliary tool for preliminary screening when experimental determination is difficult. While metastable phase formation during rapid solidification is strongly dictated by non‐equilibrium kinetics, as exemplified by the metastable eutectic phenomenon near Cu_50_Zr_50_ [16], exhaustively accounting for such transient phases would introduce prohibitive complexity. For instance, varying cooling pathways can trigger diverse competing crystallization behaviors [49, 50], which severely undermine the predictive power required for broad compositional screening. Therefore, the present framework deliberately targets equilibrium competing phases from binary diagrams. This provides a robust and reproducible thermodynamic baseline to capture the dominant phase‐competition tendencies. In the context of the Cu‐Zr system, this selection is physically substantiated by in situ undercooling experiments confirming that B2‐CuZr acts as the invariant primary phase across wide undercooling regimes [51], solidifying its status as the definitive competing phase for glass formation.

While Equation (1) is a simplification of the rigorous ternary expression that would incorporate high‐order phases (i.e., *α*Δ*S*_m_^A‐B^ = *β*Δ*S*_m_^A‐C^ = *γ*Δ*S*_m_^B‐C^ = *φ*Δ*S*_m_^A‐B‐C^, where the sum of weight factors equals 1), this treatment is physically justified in the Cu‐Zr‐Ti system. The Zr‐Ti pair forms an infinite solid solution with zero mixing enthalpy, which is highly prone to precipitation from the melt and highly detrimental to glass formation. Similarly, the ternary Laves phase Cu_2_ZrTi exists over a wide compositional range and is also highly prone to precipitate, severely restricting the glass‐forming composition window [52]. In contrast, owing to composition fluctuations and atomic migration, the crystallization of compounds and eutectic phases is more difficult than that of elements and solid solutions, thereby leaving greater opportunity for glass formation [53]. Therefore, this study focuses only on the binary Cu‐Zr and Cu‐Ti phases. Notably, ternary phases with low melting entropy should also be considered for locating optimal glass‐forming compositions. Our recent quaternary Zr‐Ti‐Ni‐Be study confirmed that incorporating such phases yields significantly enhanced glass‐forming ability compared with considering only binary phases [54].

**FIGURE 1:**
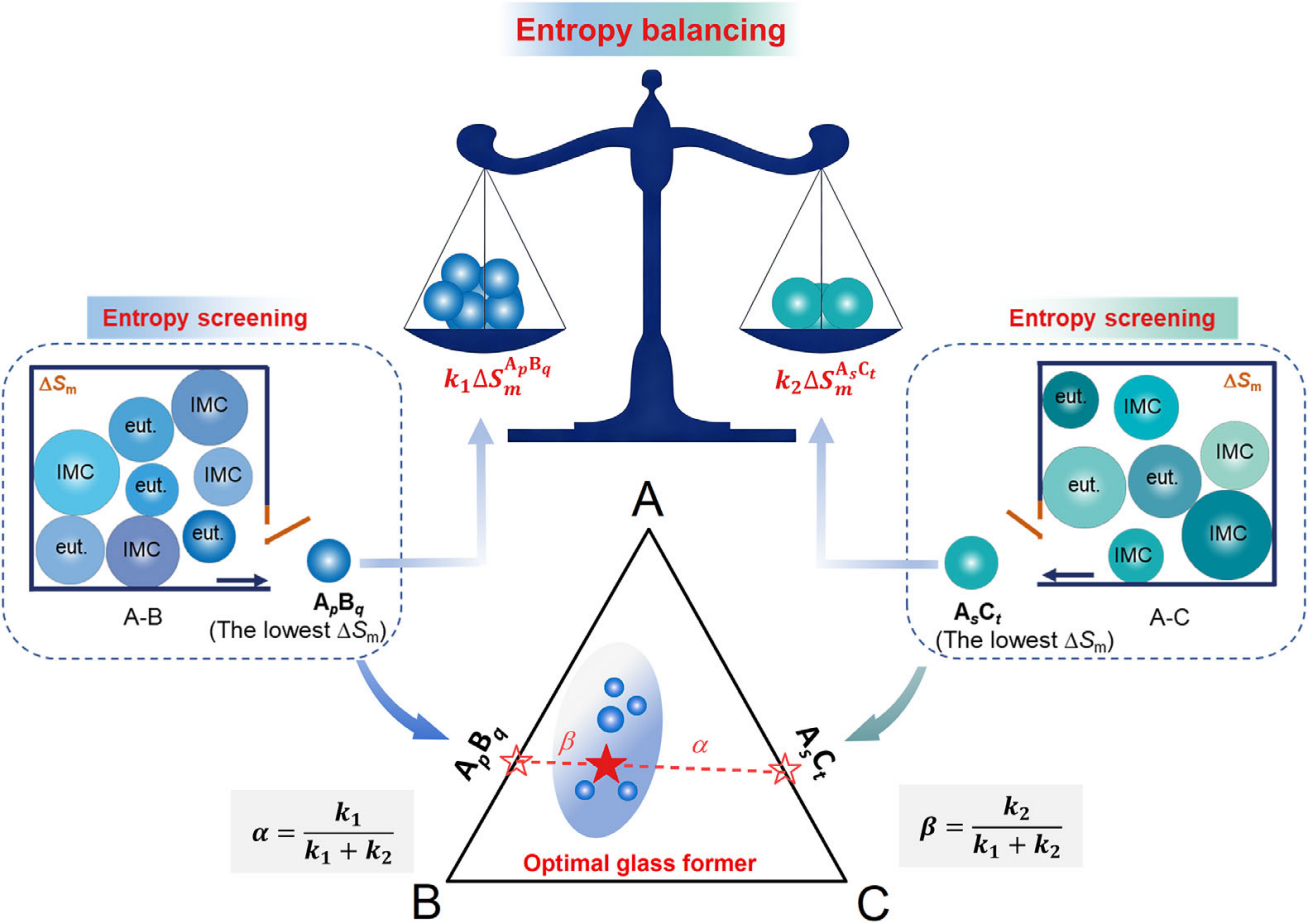
Schematic of composition design strategy for metallic glasses driven by the entropy engineering for the three‐component alloys, covering entropy screening to determine the initial phases in two binary A‐B and A‐C alloys in terms of their melting entropies and subsequent entropy balancing to achieve the phase combination of the individual phases in terms of specific percentage. The red pentagram in the A‐B‐C triangle indicates the composition predicted by entropy engineering, while the blue spheres represent compositions by trial‐and‐error. The shaded ellipse denotes the glass‐forming region.

Based on this entropy‐strategy, we first conducted experimental studies on the thermodynamics and GFA of relevant binary eutectics and intermetallic compounds in the Cu‐Zr and Cu‐Ti systems. Subsequently, ternary alloys were designed by applying the entropy‐weighted lever rule as reflected by Equation (1) to these binary phases with different Δ*S*_m_ values and GFA. This design workflow is schematically illustrated in Figure 1, which highlights the critical role of Δ*S*_m_ in both phase‐selection and phase‐balancing stages. Using a ternary phase diagram as an example, the solid pentagram denotes a composition designed by entropy engineering, derived from low‐Δ*S*_m_ initial phases (open pentagons). For comparison, blue spheres represent compositions typically explored through the traditional enthalpy‐controlled method.

## 3 Results

**FIGURE 2:**
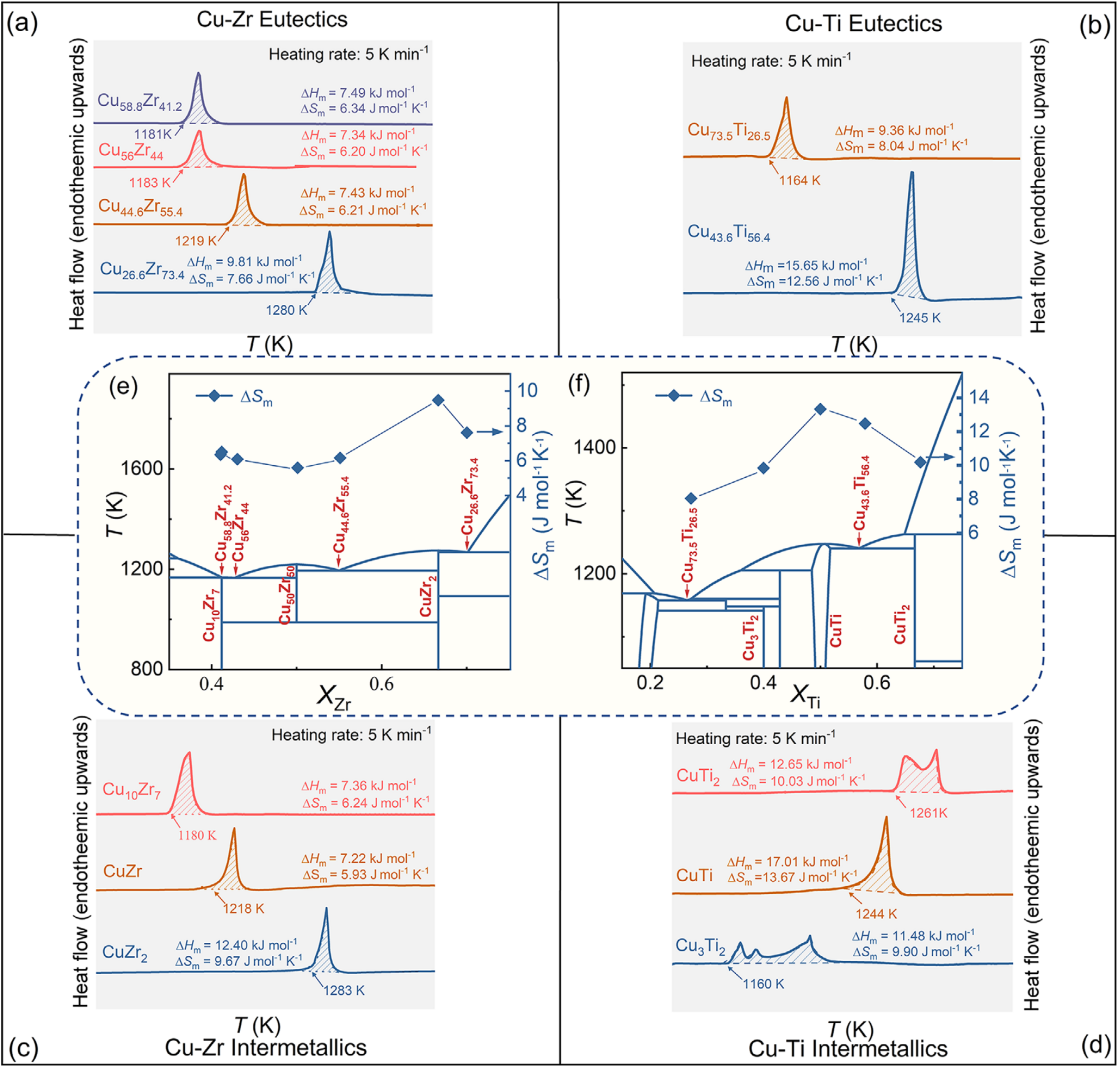
Thermodynamic characterization of eutectic and intermetallic phases in Cu‐Zr and Cu‐Ti alloys. (a–d) Experimental DSC melting curves. (e,f) Composition dependence of the melting entropy (Δ*S*_m_) for phases in the (e) Cu‐Zr and (f) Cu‐Ti binary phase diagrams. The binary phase diagrams are from literature [13, 55].

The DSC melting curves of the selected eutectic and intermetallic compositions in the Cu‐Zr and Cu‐Ti alloys are shown in Figure 2a–d. The investigated alloys include Cu‐Zr [Cu_58.8_Zr_41.2_, Cu_56_Zr_44_, Cu_44.6_Zr_55.4_, Cu_26.6_Zr_73.4_ (eut.); Cu_50_Zr_50_, Cu_10_Zr_7_, CuZr_2_ (IMC)] and Cu‐Ti [Cu_73.5_Ti_26.5_, Cu_43.6_Ti_56.4_ (eut.); Cu_3_Ti_2_, Cu_50_Ti_50_, CuTi_2_ (IMC)]. The composition‐dependent Δ*S*_m_ of the corresponding phases in the two binary alloys is summarized in Figure 2e,f, the phase diagrams are adapted from literature [13, 55]. DSC was performed at a heating rate of 5 K min^−1^ to characterize the melting behavior of the selected compositions, as shown in Figure 2a–d. The onset melting temperature, *T*_m_, was defined as the melting point, while the melting enthalpy, Δ*H*_m_ was determined from the integrated area under the endothermic peak. The Δ*S*_m_ was calculated using the formula Δ*S*_m_ = Δ*H*_m_ *T*_m_^−1^. Except for the intermetallic compounds Cu_3_Ti_2_ and CuTi_2_, all examined compositions exhibit a single endothermic peak during melting, indicating a congruent melting. The measured *T*_m_ values agreed well with the reported data, confirming that the experimental compositions matched their nominal composition. For alloys containing multiple endothermic peaks, melting occurred within a certain temperature range rather than at a specific temperature, which leads to the ambiguity of the physical meaning of the Δ*S*_m_ result calculated by the formula Δ*S*_m_ = Δ*H*_m_ *T*_m_^−1^. Nevertheless, for the purpose of conducting comparative studies, the onset melting temperature was taken as *T*_m_, while the total melting enthalpy was used to estimate ΔS_m_. The measured *T*_m_, Δ*H*_m_, and Δ*S*_m_ values are summarized in Table 1.

**TABLE 1:** Experimentally measured thermodynamic parameters of melting points *T*_m_, melting enthalpy Δ*H*_m_, and melting entropy Δ*S*_m_ together with critical copper‐wheel speed *R*_v‐c_ for eutectic (eut.) and intermetallic (IMC) phases in the Cu‐Zr and Cu‐Ti alloys.

Alloys (IMC or eut.)	*T*_m_ (K)	Δ*H*_m_ (kJ mol^−1^)	Δ*S*_m_ (J mol^−1^ K^−1^)	GFA *R*_v‐c_ (m s^−1^)
Cu_56_Zr_44_ (eut.)	1183	7.34	6.20	1 (20)
Cu_58.8_Zr_41.2_ (eut.)	1181	7.49	6.34	2 (25)
Cu_44.6_Zr_55.4_ (eut.)	1219	7.43	6.21	2 (25)
Cu_26.6_Zr_73.4_ (eut.)	1280	9.81	7.66	3 (50)
Cu_50_Zr_50_ (IMC)	1218	7.22	5.93	1 (10)
Cu_10_Zr_7_ (IMC)	1178	7.35	6.24	2 (25)
CuZr_2_ (IMC)	1283	12.4	9.67	3 (45)
Cu_73.5_Ti_26.5_ (eut.)	1164	9.36	8.04	1 (40)
Cu_43.6_Ti_56.4_ (eut.)	1245	15.65	12.56	2 (>60)
Cu_3_Ti_2_ (IMC)	1160	11.48	9.90	1 (60)
CuTi_2_ (IMC)	1261	12.65	10.03	2 (>60)
Cu_50_Ti_50_ (IMC)	1244	17.01	13.67	2 (>60)

**FIGURE 3:**
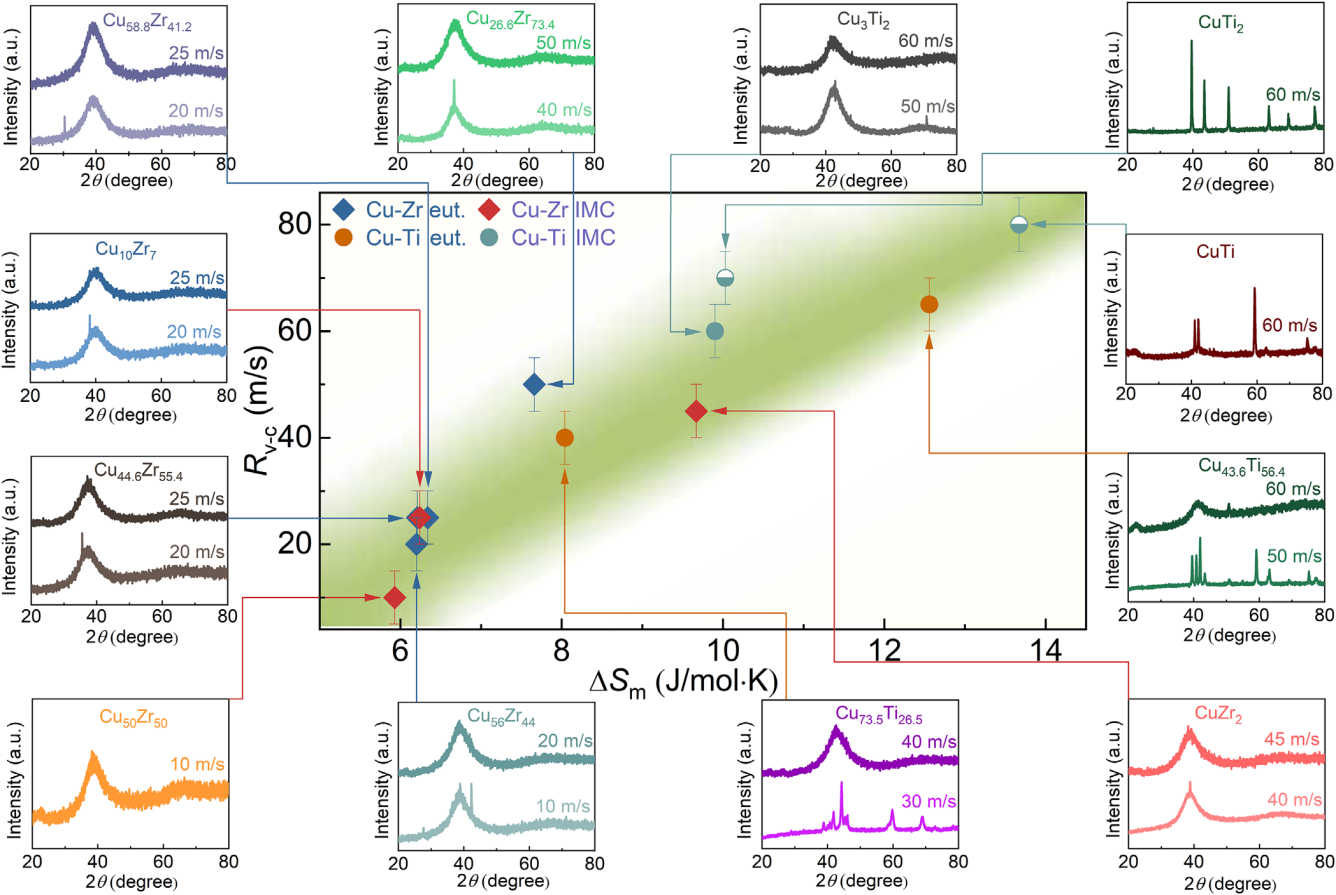
XRD patterns of Cu‐Zr and Cu‐Ti eutectic and intermetallic ribbons melt‐spun at various copper‐wheel speeds, combined with the correlation between the critical copper‐wheel speed (*R*_v‐c_) and melting entropy (Δ*S*_m_). Solid symbols represent the alloys experimentally fully transformable into MGs, and half‐filled symbols indicate the results extrapolated from the crystallinity for the alloy not fully transformable into MGs.

Figure 3 shows the XRD patterns of the as‐spun ribbons of the eutectic and intermetallic compositions of the Cu‐Zr and Cu‐Ti alloys, along with the correlation between the Δ*S*_m_ and the critical copper‐wheel speed (*R*_v‐c_, the minimum velocity required for preparing full glass formation). In the Cu‐Zr alloys, the XRD results obtained at different copper wheel speeds indicate that the *R*_v‐c_ values for the eutectic alloys Cu_58.8_Zr_41.2_, Cu_56_Zr_44_, Cu_44.6_Zr_55.4_, and Cu_26.6_Zr_73.4_ are approximately 20, 20, 25, and 50 m s^−1^, respectively, while the *R*_v‐c_ values of the intermetallic phases Cu_50_Zr_50_, Cu_10_Zr_7_, and CuZr_2_ are approximately 10, 25, and 45 m s^−1^, respectively. These results suggest that the intermetallic compound of Cu_50_Zr_50_ possesses the highest GFA among the tested Cu‐Zr compositions. In the Cu‐Ti alloys, the eutectic alloy Cu_73.5_Ti_26.5_ has the lowest *R*_v‐c_ value of 40 m s^−1^, followed by Cu_3_Ti_2_ at approximately 60 m s^−1^. The appearance of distinct crystalline peaks in the XRD patterns indicates that the *R*_v‐c_ values of other Cu‐Ti alloy exceed 60 m s^−1^, which represents the upper limit of our experimental apparatus. Overall, the GFA of the Cu‐Ti alloys decreases in the order, Cu_73.5_Ti_26.5_> Cu_3_Ti_2_> Cu_43.6_Ti_56.4_> Cu_50_Ti_50_. Notably, in both binary alloys, the optimal glass‐forming compositions are Cu_50_Zr_50_ and Cu_73.5_Ti_26.5_, both of which possess the lowest Δ*S*_m_ and the highest GFA, indicating that a low‐Δ*S*_m_ favors glass formation.

The relationship between Δ*S*_m_ and *R*_v‐c_, derived from experimental data across both Zr‐Cu and Ti‐Cu alloys, shows a clear trend that alloys with lower Δ*S*_m_ generally correspond to lower *R*_v‐c_ and higher GFA, although there is some deviation in the data. The observed date deviation could be attributed to errors in the experimental values of *R*_v‐c_ and Δ*S*_m_. In particular, the *R*_v‐c_ values for fully glass‐forming materials are often overestimated due to instrumental limitations, while those for partially crystalline alloys (e.g., Cu_50_Ti_50_ and CuTi_2_) are underestimated since 60 m s^−1^ represents the maximum attainable speed in our setup. There are also experimental errors in the measured data of Δ*S*_m_, especially in alloys where the DSC traces contain multiple endothermic peaks. Nevertheless, the intrinsic correlation between Δ*S*_m_ and GFA remains robust. This consistent relationship confirms that Δ*S*_m_ serves as a reliable and fundamental parameter for designing MGs, in agreement with our previous research [56, 57].

**FIGURE 4:**
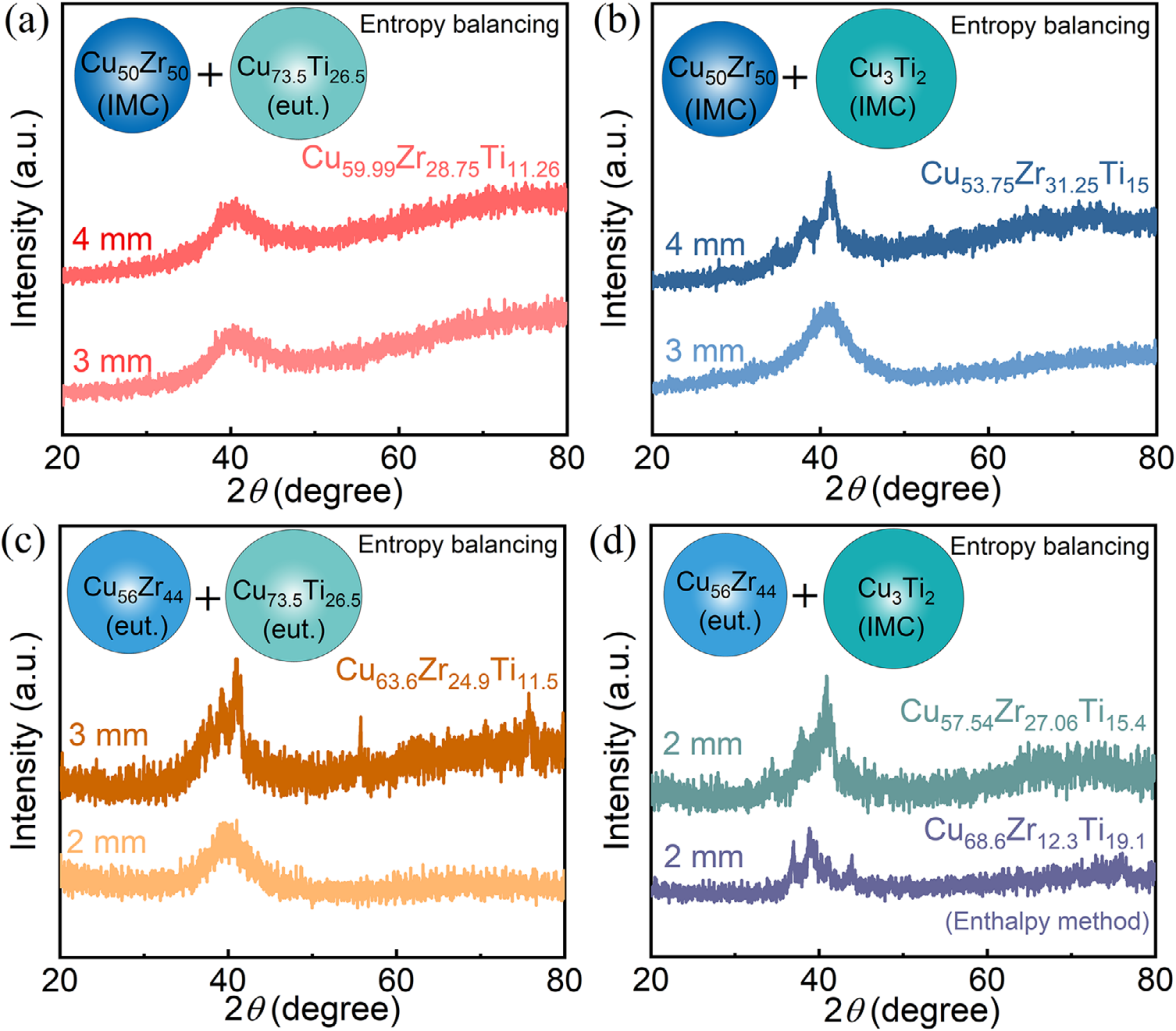
XRD patterns of suction‐cast Cu‐Zr‐Ti alloys designed by phase combinations using four initial phases shown in the central spheres. The purple components in (d) originate from an enthalpy‐based design, whereas the others are derived from entropy‐engineering design. Differences in glass‐forming ability is addressed by the diameters of glassy rods with the designed compositions in this study.

To investigate the effects of initial phase selection and entropy engineering on phase competition, four ternary compositions were designed in the Cu‐Zr‐Ti alloys. These alloys were developed by combining binary eutectic and intermetallic phases from the Cu‐Zr and Cu‐Ti alloys with relatively low‐Δ*S*_m_. The selected initial phases were Cu_50_Zr_50_ (Δ*S*_m_ = 5.93 J mol^−1^ K^−1^), Cu_56_Zr_44_ (Δ*S*_m_ = 6.2 J mol^−1^ K^−1^), Cu_73.5_Ti_26.5_ (Δ*S*_m_ = 8.04 J mol^−1^ K^−1^), and Cu_3_Ti_2_ (Δ*S*_m_ = 9.9 J mol^−1^ K^−1^). Phases from the Zr‐Ti alloys were excluded because the infinite miscibility of Zr and Ti facilitates solid‐solution crystallization, thereby reducing phase competition. Based on these initial phases, four alloys were designed via entropy engineering: Cu_57.54_Zr_27.06_Ti_15.4_ (Cu_56_Zr_44_ + Cu_3_Ti_2_), Cu_63.6_Zr_24.9_Ti_11.5_ (Cu_56_Zr_44_ + Cu_73.5_Ti_26.5_), Cu_53.75_Zr_31.25_Ti_15_ (Cu_50_Zr_50_ + Cu_3_Ti_2_), and Cu_59.99_Zr_28.75_Ti_11.26_ (Cu_50_Zr_50_ + Cu_73.5_Ti_26.5_). For comparison, an additional alloy, Cu_68.6_Zr_12.3_Ti_19.1_, was fabricated using a composition obtained by Lu's method from the binary eutectics of Cu_56_Zr_44_ and Cu_73.5_Ti_26.5_ [58]. The as‐cast rods were prepared using copper mold casting, and their structural features were examined by XRD as shown in Figure 4. The sharp crystalline peaks present in XRD patterns of the as‐cast alloys of Cu_68.6_Zr_12.3_Ti_19.1_ and Cu_57.54_Zr_27.06_Ti_15.4_ with a diameter of 2 mm, indicate their critical diameters (*D*_c_) are much lower than 2 mm. In comparison, Cu_63.6_Zr_24.9_Ti_11.5_ showed broader amorphous halos with weaker crystalline peaks, corresponding to a *D*_c_ slightly above 2 mm. Cu_53.75_Zr_31.25_Ti_15_ exhibited a *D*_c_ value between 3 and 4 mm, while the *D*_c_ value of Cu_59.99_Zr_28.75_Ti_11.26_ is well 4 mm as reflected by the fully amorphous diffraction patterns, demonstrating the highest GFA among the designed alloys.

**FIGURE 5:**
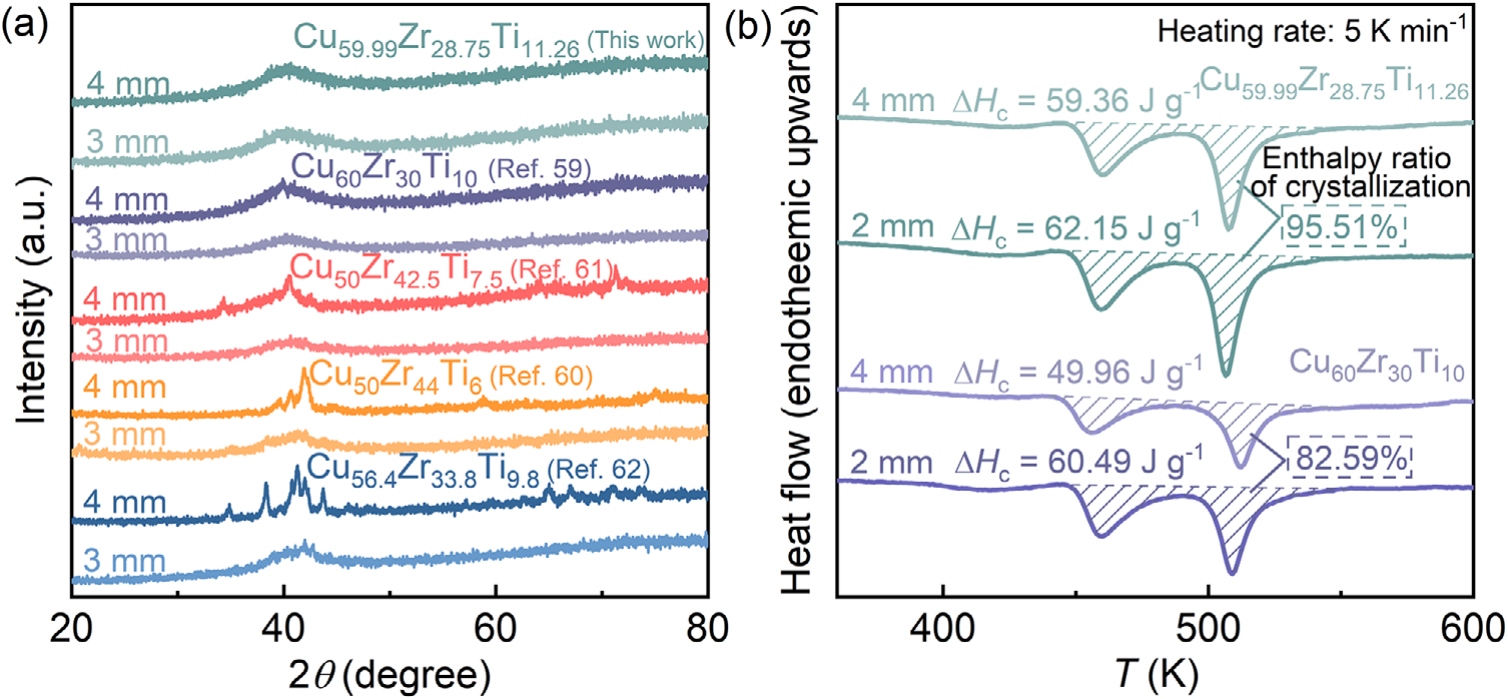
Comparison of glass‐forming ability between the Cu_59.99_Zr_28.75_Ti_11.26_ composition designed in this study and previously reported Cu‐Zr‐Ti alloys. (a) XRD patterns of the present and reported compositions. (b) Up‐scanned DSC curves of the present composition and the reported optimal composition of Cu_60_Zr_30_Ti_10_. The samples are cut from cast rods with diameters of 2 and 4 mm.

To further evaluate the GFA enhancement achieved by entropy engineering, Figure 5a compares the designed composition Cu_59.99_Zr_28.75_Ti_11.26_ with previously reported Cu‐Zr‐Ti glass formers [59, 60, 61, 62]. The XRD patterns of Cu_59.99_Zr_28.75_Ti_11.26_ and the reported compositions of Cu_60_Zr_30_Ti_10_ [59], Cu_50_Zr_44_Ti_6_ [60], Cu_50_Zr_42.5_Ti_7.5_ [61], Cu_56.4_Zr_33.8_Ti_9.8_ [62], were examined under identical conditions. All these alloys formed fully glassy structures in 3 mm rods, while partial crystallization occurred at 4 mm. In contrast, in the XRD patterns of the as‐cast alloy of Cu_60_Zr_30_Ti_10_ with a diameter of 4 mm, only weak crystalline peaks can be detected to be superimposed on a broad amorphous halo, consistent with its previously reported high GFA. Although Cu_50_Zr_42.5_Ti_7.5_ and Cu_56.4_Zr_33.8_Ti_9.8_ were reported to have a maximum critical diameter up to 5 mm, only 3 mm glassy rods were obtained under the present casting conditions, and clear crystalline peaks emerged at 4 mm. The measured critical diameter *D*_c_ for the designed and reference compositions are summarized in Table 2. Naturally, the designed alloy Cu_59.99_Zr_28.75_Ti_11.26_ exhibited higher GFA than the reported optimal composition Cu_60_Zr_30_Ti_10_. Figure 5b compares the heating DSC traces for the Cu_59.99_Zr_28.75_Ti_11.26_ and the reported Cu_60_Zr_30_Ti_10_ glassy alloys prepared in both 2 and 4 mm diameter rods. The 2 mm fully glassy samples were used as references to determine the standard crystallization enthalpy, which was then applied to quantify the glass fraction in the 4 mm samples. It was found that in the as‐cast rods with a diameter of 4 mm, the calculated glass fraction in the rod of Cu_59.99_Zr_28.75_Ti_11.26_ reached 95.51%, indicating a nearly fully glassy structure, whereas the glass fraction in the rod of reported Cu_60_Zr_30_Ti_10_ showed a lower value of 82.59%. Given the close proximity of their compositions, this result demonstrates that entropy engineering successfully optimized the previously reported composition.

**TABLE 2:** Comparison of glass‐forming compositions designed in this study and those reported in the literature, along with their corresponding critical diameter *D*_c_.

Initial phases or their combination	Compositions	Design methods	*D*_c_ (mm)
0.281(Cu_56_Zr_44_) + 0.719(Cu_73.5_Ti_26.5_)	Cu_68.6_Zr_12.3_Ti_19.1_	Δ*H*_mix_ [58]	<2
0.615(Cu_56_Zr_44_) + 0.385(Cu_3_Ti_2_)	Cu_57.54_Zr_27.06_Ti_15.4_	Δ*S*_m_	<2
0.565(Cu_56_Zr_44_) + 0.435(Cu_73.5_Ti_26.5_)	Cu_63.6_Zr_24.9_Ti_11.5_	Δ*S*_m_	2
0.625(Cu_50_Zr_50_) + 0.375(Cu_3_Ti_2_)	Cu_53.75_Zr_31.25_Ti_15_	Δ*S*_m_	3
0.575(Cu_50_Zr_50_) + 0.425(Cu_73.5_Ti_26.5_)	Cu_59.99_Zr_28.75_Ti_11.26_	Δ*S*_m_	4
Cu_60_Zr_40‐x_Ti_x_	Cu_60_Zr_30_Ti_10_	Microalloying [59]	≈4
—	Cu_50_Zr_44_Ti_6_	Thermodynamic [60]	3
Cu_50_Zr_50‐x_Ti_x_	Cu_50_Zr_42.5_Ti_7.5_	Microalloying [61]	3
—	Cu_56.4_Zr_33.8_Ti_9.8_	Microalloying [62]	3

**FIGURE 6:**
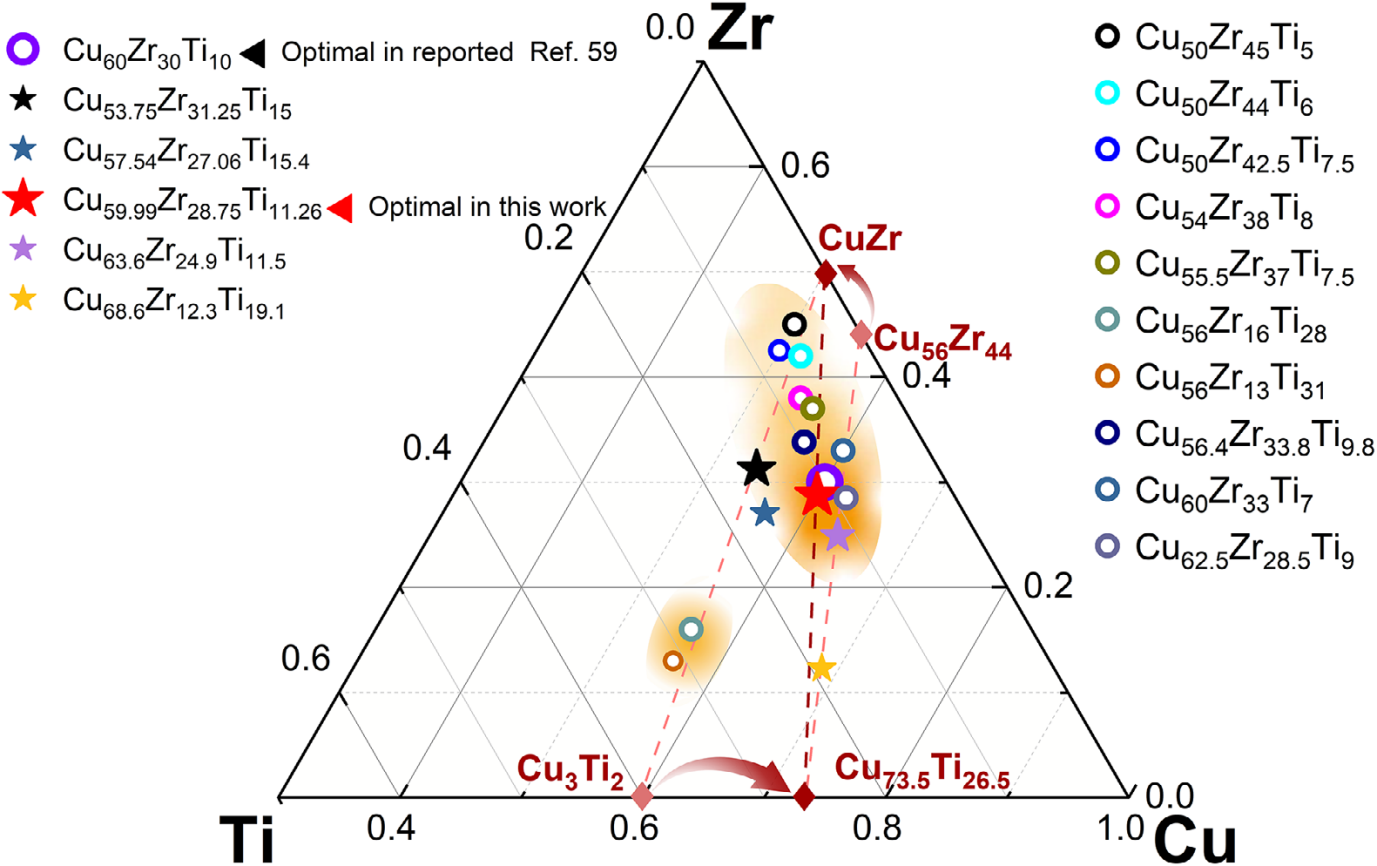
Composition diagram of ternary Cu‐Zr‐Ti metallic glasses designed via different approaches. Solid pentagrams represent the compositions designed in this study, with the optimal one shown by the red solid pentagram. Open circles denote previously reported compositions, and the large purple circle indicates the reported optimal glass‐forming composition. Diamonds along the edges correspond to the initial binary alloy compositions.

Figure 6 shows a composition map of the Cu‐Zr‐Ti ternary alloys, enabling an intuitive comparison between the reported and newly designed alloys. The reported compositions (open symbols) are mainly located in the molar composition range of 50–65 mol.% Cu, overlapping with the optimal glass‐forming region of the binary Cu‐Zr alloys. The Solid pentagrams represent the newly designed compositions obtained using entropy engineering and Lu's method. The composition designed using Lu's method (yellow pentagrams) lies outside the main glass‐forming region, consistent with its relatively poor GFA. In comparison, the four alloys derived from entropy engineering (solid pentagrams) are located within the optimal 50–65 mol.% Cu region. Remarkably, the new composition Cu_59.99_Zr_28.75_Ti_11.26_ (red pentagrams) nearly coincides with the previously reported optimal composition Cu_60_Zr_30_Ti_10_ (purple circle). This convergence indicates that entropy engineering driven by Δ*S*_m_ converges to the same endpoint in searching the optimal glass‐forming composition, obtained by doing the traditional empirical method, but with substantially higher design efficiency.

## 4 Discussion

### 4.1 Validity and Reliability of Entropy Engineering for Glass Formation

The role of Δ*S*_m_ in crystalline materials during glass formation has been elucidated from multiple perspectives, including the thermodynamics of liquid and crystalline phases [18, 63, 64, 65], the liquid–solid Gibbs free energy difference [23, 66], and crystal growth kinetics [47, 67]. In particular, our recent studies of thermodynamic and kinetic correlation in metallic alloys with regard to glass formation further revealed that if a crystal has a lower Δ*S*_m_, upon melting, higher melting viscosity can be expected, which kinetically favors glass formation [68]. Together, these studies underscore the pivotal role of Δ*S*_m_ in governing glass formation.

The low‐Δ*S*_m_ thermodynamic feature of glass‐forming crystalline materials aligns with Angell's concept of “barely stable compounds” [69], which argues that crystalline phases prone to glass formation typically display poor packing density, low lattice energy, weak crystallization tendency, and low melting points. Consistent with this view, we find that binary alloys with high GFA are characterized by both low‐Δ*S*_m_ and high vibrational entropy [70]. However, we also clarify that alloys with low‐Δ*S*_m_ do not necessarily exhibit low melting points [45], highlighting an extension of Angell's qualitative model into a quantitative thermodynamic framework.

Entropy engineering for metallic glass design consists of two key steps, initial entropy screening and subsequent entropy balancing. Initially, binary phases with the lowest Δ*S*_m_ are selected as the starting points. Subsequently, these phases are combined according to their mole fractions to achieve an overall thermodynamic entropy balance. For example, in the Cu‐Zr and Cu‐Ti alloys, the intermetallic Cu_50_Zr_50_ and eutectic Cu_73.5_Ti_26.5_ phases exhibit the lowest Δ*S*_m_ and highest GFA, confirming that low‐Δ*S*_m_ phases are more prone to glass formation. Building on this principle, the ternary Cu‐Zr‐Ti alloy Cu_59.99_Zr_28.75_Ti_11.26_ was designed by using intermetallic Cu_50_Zr_50_ and the eutectic Cu_73.5_Ti_26.5_ phases as the initial phases, demonstrating superior GFA. It also shows remarkable agreement with the reported optimal composition, Cu_60_Zr_30_Ti_10_ [59]. Similarly, when using the two deep eutectic phases (Cu_56_Zr_44_ and Cu_73.5_Ti_26.5_), entropy balancing produced Cu_63.6_Zr_24.9_Ti_11.5_, which achieved a larger *D*_c_ than the composition Cu_68.6_Zr_12.3_Ti_19.1_ obtained via the mixed‐enthalpy method.

This performance discrepancy stems from the semi‐empirical nature of the Miedema method for calculating Δ*H*_mix_, which yields approximate predictions that do not correlate monotonically with GFA. To rigorously verify the superiority of the entropy‐based approach, we expanded our quantitative statistical analysis to encompass multiple binary alloy systems (Cu‐Zr, Cu‐Ti, Zr‐Ni, Zr‐Co, and Hf‐Cu) [57, 68]. As detailed in Figure S2, Δ*S*_m_ exhibits a strict monotonic correlation with GFA across these diverse compositions. In contrast, classical empirical parameters, such as Δ*H*_mix_ and mismatch entropy (*S*_σ_/*K_b_*), frequently display scattered and non‐monotonic relationships with GFA indicators (Figure S2b,c). This indicates that, compared to Δ*H*_mix_ and S_σ_/*k_b_*, Δ*S_m_* can provide a more comprehensive assessment of glass formation. It should be noted that this comparison does not mean that Δ*H*_mix_ and (*S*_σ_/*K_b_*) is not significant for glass formation. The roles of Δ*H*_mix_ and (*S*_σ_/*K_b_*) in glass formation may be incorporated into the value of the melting entropy, as implied by the previously reported studies [71, 72]. Furthermore, this Δ*S*_m_‐dominated trend is corroborated by thermodynamic analyses of classical bulk metallic glasses, where low‐Δ*S*_m_ systems (e.g., Vitreloy 1) consistently possess higher GFA than moderate‐Δ*S*_m_ systems (e.g., Mg‐based alloys) [21, 22]. Therefore, as an intrinsic thermodynamic property directly measurable from experiments, Δ*S*_m_ overcomes the empirical limitations of traditional descriptors, serving as a more reliable and physically meaningful weighting factor for phase evaluating and balancing.

Beyond its thermodynamic reliability, the quantitative correlation is supported by the natural linking of thermodynamics (melting entropy) and the liquid kinetics (fragility and liquid). Specifically, Δ*S*_m_ quantitatively governs the liquid fragility parameter (*m*), a key kinetic indicator for GFA, through the relation *m* = 40 Δ*C*_p_ (*T*_g_) Δ*S*_m_^−1^ [73]. Moreover, a quantitative cross‐system formula correlating reduced Δ*S*_m_ with melt viscosity near the melting point has been firmly established [68]. In the present Cu‐Zr and Cu‐Ti systems, this theoretical framework translates to a strictly monotonic quantitative relationship between Δ*S*_m_ and the *R*_v‐c_ (Table 1 and Figure 3). Notably, this quantitative validity extends far beyond metallic alloys, it is statistically corroborated by a substantial database encompassing diverse glass formers, including network formers, aromatic organics, alcohols, and chalcogenides. Collectively, this evidence confirms that low‐Δ*S*_m_ fundamentally governs the kinetic bottleneck for glass formation across entirely different material classes [23, 68, 73]. These cross‐system findings underscore the robust predictive potential of Δ*S*_m_ and motivate the future development of an expanded Δ*S*_m_ database, potentially integrated with machine learning, to further enhance compositional design efficiency.

### 4.2 Interpreting the Phase Competition Mechanism Using Entropy Engineering

The significance of entropy engineering is further validated by showing its ability to rank the glass formability of mixed phases, which assist in categorizing phase combinations, which is in nature, phase competition upon glass formation. The four alloys designed using entropy engineering can be ranked in a descending GFA order as follows, Cu_59.99_Zr_28.75_Ti_11.26_> Cu_53.75_Zr_31.25_Ti_15_> Cu_63.6_Zr_24.9_Ti_11.5_> Cu_57.54_Zr_27.06_Ti_15.4_. Experimental results indicate that the characteristics of the initial binary phases exert a direct influence on the GFA of the designed compositions. Alloys designed from phases with low‐Δ*S*_m_ consistently exhibit higher GFA than those designed from phases with high‐Δ*S*_m_. For instance, Cu_59.99_Zr_28.75_Ti_11.26_, designed from the lowest Δ*S*_m_ phases (Cu_50_Zr_50_ and Cu_73.5_Ti_26.5_), displays the optimal GFA, even surpassing the previously reported Cu_60_Zr_30_Ti_10_. Similarly, the Cu_53.75_Zr_31.25_Ti_15_ (derived from Cu_50_Zr_50_ + Cu_3_Ti_2_) and Cu_63.6_Zr_24.9_Ti_11.5_ (Cu_56_Zr_44_ + Cu_73.5_Ti_26.5_) alloys, each designed from a combination of one low‐Δ*S*_m_ initial phase and one high‐Δ*S*_m_ initial phase, correspond precisely to the second and third highest GFA values. In contrast, the Cu_57.54_Zr_27.06_Ti_15.4_ alloy, designed by combining two high‐Δ*S*_m_ phases, exhibits the lowest GFA among the designed compositions.

This experimental evidence demonstrates that phase competition among low‐Δ*S*_m_ phases under entropy balancing reduces the crystallization tendency, thereby promoting glass formation. It thus quantitatively answers the fundamental question regarding the correlation between phase competition and glass formation, and confirms that competition among low‐Δ*S*_m_ initial phases is more conducive to glass formation than that of high‐Δ*S*_m_ initial phases. More importantly, these findings underscore the significance of entropy engineering in controlling phase competition during solidification [55, 56, 71, 72, 73, 74, 75, 76].

Earlier studies primarily attributed glass formation to the frustration of nucleation and growth among competing crystalline phases [38, 77, 78], emphasizing energetic, chemical, or topological descriptors of these phases [39, 79, 80]. In addition to such descriptors, mechanistic studies have shown that the mutual interference among competing phases reduces their crystallization tendency, suppresses long‐range atomic ordering, and thereby enhances GFA [81]. While such descriptors provide valuable qualitative insights, a quantitative understanding has remained elusive. By contrast, introducing Δ*S*_m_ as a quantifiable thermodynamic parameter enables a clear and quantitative interpretation of (1) how to sort out the phases in order to achieve enhanced GFA, (2) how the competition proceeds when knowing which phases are engaged in competition for a specific alloy composition upon precipitation.

Compared with conventional design strategies, entropy engineering provides distinct advantages. Traditional trial‐and‐error microalloying often yields scattered optimal compositions (see Figure 6), while enthalpy‐balancing approaches frequently deviate from the true optimum [58]. For example, in the Cu‐Zr alloys, the optimal glass‐forming composition Cu_50_Zr_50_ is intermetallic rather than eutectic [15, 82]. Similarly, alloy‐design attempts based on intermetallic compounds did not fully recognize the underlying thermodynamic attributes that govern glass formation [83]. By contrast, entropy engineering directly and quantitatively regulates phase competition and reduces crystallization tendency during solidification based on the quantitative principle of low‐Δ*S*_m_. The investigation of Cu‐Zr‐Ti alloys verifies this strategy, demonstrating that selecting initial phases with low‐Δ*S*_m_ significantly enhances GFA. This establishes a predictive framework for the rational optimization of GFA through strategic selection of initial phases and deliberate regulation of phase competition, providing the first quantitative description of how entropy‐controlled phase competition governs metallic glass formation.

## 5 Conclusions

This study systematically investigates the glass formation behavior of the Cu‐Zr‐Ti alloys and reconfirms the strong correlation between melting entropy (Δ*S*_m_) and glass‐forming ability, thereby establishing entropy engineering as a fundamental and efficient framework for metallic glass composition design. On this basis, the study quantitatively elucidates, for the first time, the role of phase competition in glass formation. The results demonstrate that low‐Δ*S*_m_ is a key characteristic of competing phases conducive to glass formation. By employing the Δ*S*_m_ of individual phases as a thermodynamic weighting factor, entropy engineering enables quantitative regulation of phase competition and effectively suppresses crystallization. These findings provide a mechanistic foundation for entropy‐engineering‐guided alloy design and offer new insights into the fundamental mechanisms governing metallic glass formation.

## 6 Materials and Methods

Homogeneous alloy ingots were melted five times under argon protection in a vacuum arc furnace, using high‐purity copper (99.99%), zirconium (99.99%), and titanium (99.99%). Prior to melting, the raw metal materials were surface‐treated to remove oxidation films and impurities. As‐spun ribbons of Cu‐Zr and Cu‐Ti alloys were prepared using the melt‐spinning method at different wheel velocities. The alloy ingots of Cu‐Zr‐Ti were cast into alloy rods with diameters of 2, 3, and 4 mm using the suction casting method. The structural nature of the rods and strips was characterized by X‐ray diffraction (XRD) using a Shimadzu diffractometer equipped with Cu‐Kα radiation. The melting enthalpies and melting points of the alloys were measured using a Netzsch STA 449C differential scanning calorimeter (DSC) at a fixed heating rate of 5 K min^−1^. To ensure the homogenization of the alloy microstructure, all DSC samples were extracted the center of the homogenized and annealed master alloy ingots, and the instrument was calibrated using pure Au.

## Author Contributions

**B.K. Huo**: Writing – original draft, Methodology, Data curation, Investigation, Conceptualization. **Z.Q. Cai**: Methodology, Writing – review &editing. **B.T. Wang**: Methodology, Writing – review and editing. **Z.Q. Song**: Writing – review and editing. **H. Bo**: Writing – review and editing. **S.D. Feng**: Writing – review and editing. **Z.J. Li**: Writing – review and editing. **X.J Liu**: Writing – review and editing. **L.M. Wang**: Writing – review and editing, Validation, Supervision, Resources, Conceptualization.

## Funding

This work was supported by the National Natural Science Foundation of China (Grant Nos. 52271155, 52571197, 52271154, and 52471185), the Yanshan University Fundamental Innovation Research Cultivation Project (Grant No. 2023LGZD002), and the Hebei Yanzhao Golden Platform Talent Gathering Plan (Grant No. HY2024050013).

## Conflicts of Interest

The authors declare no conflicts of interest.

## Supporting information




**Supporting File**: advs75936‐sup‐0001‐SuppMat.docx.

## Data Availability

The data that support the findings of this study are available from the corresponding author upon reasonable request.

## References

[1] G. Liu , S. Sohn , S. A. Kube , et al., “Machine Learning Versus Human Learning in Predicting Glass‐Forming Ability of Metallic Glasses,” Acta Materialia 243 (2023): 118497, 10.1016/j.actamat.2022.118497.

[2] H. T. Bu , H. W. Luan , J. Y. Kang , et al., “Accessing Ultrastable Glass via a Bulk Transformation,” Nature Communications 16 (2025): 562, 10.1038/s41467-024-55367-8. PMC11724126 39794324

[3] J. Schroers , “Processing of Bulk Metallic Glass,” Advanced Materials 22 (2010): 1566–1597, 10.1002/adma.200902776. 20496386

[4] D. Ma , A. D. Stoica , and X. L. Wang , “Power‐Law Scaling and Fractal Nature of Medium‐Range Order in Metallic Glasses,” Nature Materials 8 (2009): 30–34, 10.1038/nmat2340. 19060888

[5] A. Hirata , P. Guan , T. Fujita , et al., “Direct Observation of Local Atomic Order in a Metallic Glass,” Nature Materials 10 (2011): 28–33, 10.1038/nmat2897. 21102454

[6] W. K. Luo , H. W. Sheng , F. M. Alamgir , J. M. Bai , J. H. He , and E. Ma , “Icosahedral Short‐Range Order in Amorphous Alloys,” Physical Review Letters 92 (2004): 145502, 10.1103/PhysRevLett.92.145502. 15089549

[7] Z. Y. Yang , Q. Miao , J. K. Dan , M. T. Liu , and Y. J. Wang , “Structural Mechanism of Glass Transition Uncovered by Unsupervised Machine Learning,” Acta Materialia 281 (2024): 120410, 10.1016/j.actamat.2024.120410.

[8] M. X. Li , Y. T. Sun , C. Wang , et al., “Data‐driven Discovery of a Universal Indicator for Metallic Glass Forming Ability,” Nature Materials 21 (2022): 165–172, 10.1038/s41563-021-01129-6. 34737454

[9] A. L. Greer , M. B. Costa , and O. S. Houghton , “Metallic Glasses,” Science 267 (2023): 1054–1061, 10.1557/s43577-023-00586-5.

[10] Z. P. Lu and C. T. Liu , “A New Glass‐Forming Ability Criterion for Bulk Metallic Glasses,” Acta Materialia 50 (2002): 3501–3512, 10.1016/S1359-6454(02)00166-0.

[11] R. J. Highmore and A. L. Greer , “Eutectics and the Formation of Amorphous Alloys,” Nature 339 (1989): 363–365, 10.1038/339363a0.

[12] S. Sohrabi , J. Fu , L. Li , et al., “Manufacturing of Metallic Glass Components: Processes, Structures and Properties,” Progress in Materials Science 144 (2024): 101283, 10.1016/j.pmatsci.2024.101283.

[13] Y. L. Liu , S. H. Liu , C. Zhang , Y. Du , J. Wang , and Y. W. Li , “Experimental Investigation and Thermodynamic Description of the Cu‐Zr System,” Journal of Phase Equilibria and Diffusion 38 (2017): 121–134, 10.1007/s11669-017-0522-2.

[14] H. Tan , Y. Zhang , D. Ma , Y. P. Feng , and Y. Li , “Optimum Glass Formation at Off‐Eutectic Composition and Its Relation to Skewed Eutectic Coupled Zone in the La Based La–Al–(Cu,Ni) pseudo Ternary System,” Acta Materialia 51 (2003): 4551–4561, 10.1016/S1359-6454(03)00291-X.

[15] Y. X. Wang , J. H. Yao , and Y. Li , “Glass Formation Adjacent to the Intermetallic Compounds in Cu‐Zr Binary System,” Journal of Materials Science & Technology 34 (2018): 605–612, 10.1016/j.jmst.2017.09.008.

[16] W. H. Wang , J. J. Lewandowski , and A. L. Greer , “Understanding the Glass‐Forming Ability of Cu_5_0Zr_5_0 Alloys in Terms of a Metastable Eutectic,” Journal of Materials Research 20 (2005): 2307–2313, 10.1557/jmr.2005.0302.

[17] O. N. Senkov and D. B. Miracle , “Effect of the Atomic Size Distribution on Glass Forming Ability of Amorphous Metallic Alloys,” Materials Research Bulletin 36 (2001): 2183–2198, 10.1016/S0025-5408(01)00715-2.

[18] A. Inoue , “Stabilization of Metallic Supercooled Liquid and Bulk Amorphous Alloys,” Acta Materialia 48 (2000): 279–306, 10.1016/S1359-6454(99)00300-6.

[19] M. F. De Oliveira , F. S. Pereira , C. Bolfarini , C. S. Kiminami , and W. J. Botta , “Topological Instability, Average Electronegativity Difference and Glass Forming Ability of Amorphous Alloys,” Intermetallics 17 (2009): 183–185, 10.1016/j.intermet.2008.09.013.

[20] C. Chattopadhyay , K. S. N. S. Idury , J. Bhatt , K. Mondal , and B. S. Murty , “Critical Evaluation of Glass Forming Ability Criteria,” Materials Science and Technology 32 (2016): 380–400, 10.6084/M9.FIGSHARE.3178588.

[21] R. Busch , W. Liu , and W. L. Johnson , “Thermodynamics and Kinetics of the Mg_65_Cu_25_Y_10_ Bulk Metallic Glass Forming Liquid,” Journal of Applied Physics 83 (1998): 4134–4141, 10.1063/1.367167.

[22] S. C. Glade , R. Busch , D. S. Lee , W. L. Johnson , R. K. Wunderlich , and H. J. Fecht , “Thermodynamics of Cu_47_Ti_34_Zr_11_Ni_8_, Zr_52.5_Cu_17.9_Ni_14.6_Al_10_Ti_5_ and Zr_57_Cu_15.4_Ni_12.6_Al_10_Nb_5_ Bulk Metallic Glass Forming Alloys,” Journal of Applied Physics 87 (2000): 7242–7248, 10.1063/1.372975.

[23] L.‐M. Wang , Y. Tian , R. Liu , and W. Wang , “A ‘Universal’ Criterion for Metallic Glass Formation,” Applied Physics Letters 100 (2012): 261913, 10.1063/1.4731881.

[24] W. K. Tu , X. Q. Li , Z. M. Chen , et al., “Glass Formability in Medium‐Sized Molecular Systems/Pharmaceuticals. I. Thermodynamics vs. kinetics,” The Journal of Chemical Physics 144 (2016): 174502, 10.1063/1.4948323. 27155640

[25] A. Takeuchi and A. Inoue , “Calculations of Mixing Enthalpy and Mismatch Entropy for Ternary Amorphous Alloys,” Materials Transactions, JIM 41 (2000): 1372–1378, 10.2320/matertrans1989.41.1372.

[26] C. A. Angell , “Formation of Glasses from Liquids and Biopolymers,” Science 267 (1995): 1924–1935, 10.1126/science.267.5206.1924. 17770101

[27] P. G. Debenedetti and F. Stillinger , “Supercooled Liquids and the Glass Transition,” Nature 410 (2001): 259–267, 10.1038/35065704. 11258381

[28] Z. L. Long , H. Q. Wei , Y. H. Ding , P. Zhang , G. Q. Xie , and A. Inoue , “A New Criterion for Predicting the Glass‐Forming Ability of Bulk Metallic Glasses,” Journal of Alloys and Compounds 475 (2009): 207–219, 10.1016/j.jallcom.2008.07.087.

[29] C. A. Angell , “Perspective on the Glass Transition,” Journal of Physics: Condensed Matter 10 (1988): 10237–10240.

[30] M. D. Ediger , C. A. Angell , and S. R. Nagel , “Supercooled Liquids and Glasses,” The Journal of Physical Chemistry 100 (1996): 13200–13212, 10.1021/jp953538d.

[31] C. Dong , Z. J. Wang , S. Zhang , et al., “Review of Structural Models for the Compositional Interpretation of Metallic Glasses,” International Materials Reviews 65 (2020): 286–296, 10.1080/09506608.2019.1638581.

[32] D. Turnbull , “Under What Conditions Can a Glass be Formed?,” Contemporary Physics 10 (1969): 473–488, 10.1080/00107516908204405.

[33] P. I. K. Onorato and D. R. Uhlmann , “Nucleating Heterogeneities and Glass Formation,” Journal of Non‐Crystalline Solids 22 (1977): 367–378, 10.1016/0022-3093(76)90066-1.

[34] W. Dong , S. Liu , Z. Wu , et al., “Evidence of Liquid‐liquid Phase Transition in Zr‐Cu‐Al Melts and Its Link to Glass‐Forming Ability,” Applied Physics Letters 127 (2025): 191903, 10.1063/5.0299273.

[35] W. Kurz and D. J. Fisher , “Dendrite Growth in Eutectic Alloys: The Coupled Zone,” International Metals Reviews 24 (1979): 177–204, 10.1179/095066079790136326.

[36] Y. B. Zhang , Z. C. Lu , L. Wang , et al., “Eutectic Growth Kinetics as an Indicator for Glass‐forming Ability,” Intermetallics 162 (2023): 108025, 10.1016/j.intermet.2023.108025.

[37] J. D. Hunt , D. T. J. Hurle , K. A. Jackson , and E. Jakeman , “On the Theory of the Stability of Lamellar Eutectics,” Metallurgical Transactions 1 (1970): 318–320, 10.1007/BF02819287.

[38] J. Russo , F. Romano , and H. Tanaka , “Glass Forming Ability in Systems with Competing Orderings,” Physical Review X 8 (2018): 021040, 10.1103/PhysRevX.8.021040.

[39] D. H. Xu , G. Duan , and W. L. Johnson , “Unusual Glass‐forming Ability of Bulk Amorphous Alloys Based on Ordinary Metal Copper,” Physical Review Letters 92 (2004): 245504, 10.1103/PhysRevLett.92.245504. 15245096

[40] E. Perim , D. Lee , Y. H. Liu , et al., “Spectral Descriptors for Bulk Metallic Glasses Based on the Thermodynamics of Competing Crystalline Phases,” Nature Communications 7 (2016): 12315, 10.1038/ncomms12315. PMC4974662 27480126

[41] S. Lan , Z. D. Wu , X. Y. Wei , et al., “Structure Origin of a Transition of Classic‐to‐avalanche Nucleation in Zr‐Cu‐Al Bulk Metallic Glasses,” Acta Materialia 149 (2018): 108–118, 10.1016/j.actamat.2018.02.028.

[42] A. L. Greer , “Confusion by Design,” Nature 366 (1993): 303–304, 10.1038/366303a0.

[43] T. Egami , “Nano‐Glass Mechanism of Bulk Metallic Glass Formation,” Materials Transactions 43 (2002): 510–517, 10.2320/matertrans.43.510.

[44] B. Cantor , I. T. Chang , P. Knight , and A. J. B. Vincent , “Microstructural Development in Equiatomic Multicomponent Alloys,” Materials Science and Engineering: A 375‐377 (2004): 213–218, 10.1016/j.msea.2003.10.257.

[45] B. K. Ren , Z. J. Li , Y. H. Zhang , S. D. Feng , and L.‐M. Wang , “New Interpretation of Glass Formation in Isomeric Substances: Shifting from Melting‐Point to Melting‐Entropy,” Advanced Science 10 (2023): 2206389, 10.1002/advs.202206389. 36792966 PMC10104644

[46] L.‐M. Wang , Y. J. Tian , and R. P. Liu , “Dependence of Glass Forming Ability on Liquid Fragility: Thermodynamics Versus Kinetics,” Applied Physics Letters 97 (2010): 181901, 10.1063/1.3506900.

[47] Q. Wang , L.‐M. Wang , M. Z. Ma , et al., “Diffusion‐controlled Crystal Growth in Deeply Undercooled Melt on Approaching the Glass Transition,” Physical Review B 83 (2011): 134101, 10.1103/PhysRevB.83.014202.

[48] B. Q. Wu , L. T. Kong , and J. F. Li , “Composition Dependence in Glass‐Forming Ability of Cu–Ag Binary Alloys,” Acta Materialia 235 (2022): 118059, 10.1016/j.actamat.2022.118059.

[49] J. E. Schawe and J. F. Löffler , “Existence of Multiple Critical Cooling Rates Which Generate Different Types of Monolithic Metallic Glass,” Nature Communications 10 (2019): 1337, 10.1038/s41467-018-07930-3. PMC6430809 30902964

[50] F. Q. Meng , S. H. Zhou , R. T. Ott , M. J. Kramer , and R. E. Napolitano , “Competitive Devitrification and Metastable Phase Selection in Amorphous Al–Sm,” Materialia 9 (2020): 100595, 10.1016/j.mtla.2020.100595.

[51] J. Gegner , O. Shuleshova , R. Kobold , et al., “In Situ Observation of the Phase Selection from the Undercooled Melt in Cu–Zr,” Journal of Alloys and Compounds 576 (2013): 232–235, 10.1016/j.jallcom.2013.04.035.

[52] P. G. Qin , H. Wang , L. G. Zhang , H. S. Liu , and Z. P. Jin , “The Isothermal Section of the Cu–Ti–Zr System at 1023K Measured with Diffusion‐Triple Approach,” Materials Science and Engineering: A 476 (2008): 83–88, 10.1016/j.msea.2007.05.065.

[53] H. Men , J. Fu , C. Ma , S. Pang , and T. Zhang , “Bulk Glass Formation in Ternary Cu‐Zr‐Ti System,” University of Science and Technology Beijing, Mineral, Metallurgy, Material 14 (2007): 19–22.

[54] B. T. Wang , L. Yang , B. Ke‐Huo , et al., “Utilizing Melting Entropy to Evaluate Glass Formation and to Develop Zr‐Ti‐Ni‐Be Metallic Glasses,” Journal of Alloys and Compounds 1041 (2025): 183909, 10.1016/j.jallcom.2025.183909.

[55] M. A. Turchanin , P. G. Agraval , and A. R. Abdulov , “Thermodynamic Assessment of the Cu‐Ti‐Zr System. I. Cu‐Ti System,” Powder Metallurgy and Metal Ceramics 47 (2008): 344–360, 10.1007/s11106-008-9026-2.

[56] J. Wang , Z. Q. Cai , Q. Qiao , et al., “A Novel Strategy with High Targeting Performance in Designing Metallic Glasses Conducted on the Light of Melting Entropy,” Journal of Non‐Crystalline Solids 614 (2023): 122405, 10.1016/j.jnoncrysol.2023.122405.

[57] Z. Q. Cai , Z. Q. Song , Y. H. Zhang , S. D. Feng , Z. J. Li , and L.‐M. Wang , “Highly Efficient Strategy of Compositional Design for Metallic Glasses Driven by Melting Entropy,” Scripta Materialia 259 (2025): 116569, 10.1016/j.scriptamat.2025.116569.

[58] Z. P. Lu , J. Shen , D. W. Xing , J. F. Sun , and C. T. Liu , “Binary Eutectic Clusters and Glass Formation in Ideal Glass‐Forming Liquids,” Applied Physics Letters 89 (2006): 2004–2007, 10.1063/1.2336597.

[59] A. Inoue , W. Zhang , T. Zhang , and K. Kurosaka , “Thermal and Mechanical Properties of Cu‐Based Cu‐Zr‐Ti Bulk Glassy Alloys,” Materials Transactions 42 (2001): 1149–1151, 10.2320/matertrans.42.1149.

[60] Y. Pan , Y. Q. Zeng , L. J. Jing , L. Zhang , and J. H. Pi , “Composition Design and Mechanical Properties of Ternary Cu–Zr–Ti Bulk Metallic Glasses,” Materials & Design 55 (2014): 773–777, 10.1016/j.matdes.2013.10.057.

[61] H. Men , S. J. Pang , and T. Zhang , “Glass‐Forming Ability and Mechanical Properties of Cu_50_Zr_50‐x_Tix Alloys,” Materials Science and Engineering: A 408 (2005): 326–329, 10.1016/j.msea.2005.08.207.

[62] Y. Pan , H. B. Cao , L. Ding , C. Zhang , and Y. A. Chang , “Novel Bulkier Copper‐Rich Ternary Metallic Glasses from Computational Thermodynamics,” Journal of Non‐Crystalline Solids 356 (2010): 2168–2171, 10.1016/j.jnoncrysol.2010.08.003.

[63] S. Mukherjee , J. Schroers , W. Johnson , and W.‐K. Rhim , “Influence of Kinetic and Thermodynamic Factors on the Glass‐Forming Ability of Zirconium‐Based Bulk Amorphous Alloys,” Physical Review Letters 94 (2005): 245501, 10.1103/PhysRevLett.94.245501.

[64] D. Turnbull , “Kinetics of Solidification of Supercooled Liquid Mercury Droplets,” The Journal of Chemical Physics 20 (1952): 411–424, 10.1063/1.1700435.

[65] X. Y. Li , H. P. Zhang , S. Lan , et al., “Observation of High‐frequency Transverse Phonons in Metallic Glasses,” Physical Review Letters 124 (2020): 225902, 10.1103/PhysRevLett.124.225902. 32567931

[66] M. H. Cohen and D. Turnbull , “Molecular Transport in Liquids and Glasses,” The Journal of Chemical Physics 31 (1959): 1164–1169, 10.1063/1.1730566.

[67] R. Busch , J. Schroers , and W. H. Wang , “Thermodynamics and Kinetics of Bulk Metallic Glass,” MRS Bulletin 32 (2007): 620–623, 10.1557/mrs2007.122.

[68] J. Wang , T. F. Cao , H. Kang , et al., “Remarkable Relation between Melting Entropy and Kinetic Viscosity in Metallic Glasses,” Journal of Alloys and Compounds 955 (2023): 170287, 10.1016/j.jallcom.2023.170287.

[69] C. A. Angell , “Forty Years of Silica Simulations. Which Way Now?,” International Journal of Applied Glass Science 6 (2015): 3–14, 10.1111/ijag.12112.

[70] H. Kang , J. Wang , Y. H. Zhang , Z. J. Li , S. D. Feng , and L.‐M. Wang , “Understanding of Glass‐Forming Ability of Zr‐Cu Alloys from the Perspective of Vibrational Entropy of Crystalline Phases,” Journal of Applied Physics 131 (2022): 215102, 10.1063/5.0093785.

[71] P. Gao , W. K. Tu , P. F. Li , and L.‐M. Wang , “Variation in Entropies of Fusion Driven by Mixing in Binary Glass Forming Eutectics,” Journal of Alloys and Compounds 736 (2018): 12–16, 10.1016/j.jallcom.2017.11.103.

[72] Y. T. Ma , Z. C. Shi , B. T. Wang , et al., “Accordance of Glass Formation Criteria: Entropic vs Structural,” The Journal of Chemical Physics 163 (2025): 104506, 10.1063/5.0284754. 40928046

[73] L.‐M. Wang , C. A. Angell , and R. Richert , “Fragility and Thermodynamics in Nonpolymeric Glass‐Forming Liquids,” The Journal of Chemical Physics 125 (2006): 074505, 10.1063/1.2244551. 16942349

[74] H. Kang , X. Ye , J. Wang , S. F. Pan , and L.‐M. Wang , “Abnormal Bonding Ways in Zr_50_Cu_50_ Metallic Glass under High Pressures,” Journal of Alloys and Compounds 780 (2019): 512–517, 10.1016/j.jallcom.2018.12.004.

[75] W. F. Wu and Y. Li , “Bulk Metallic Glass Formation near Intermetallic Composition Through Liquid Quenching,” Applied Physics Letters 95 (2009): 011906, 10.1063/1.3168411.

[76] B. T. Wang , Z. J. Li , S. D. Feng , and L.‐M. Wang , “Design Strategy for Al‐Containing Metallic Glasses by Entropy Engineering and Covalent Attribute,” Materials & Design 252 (2025): 113771, 10.1016/j.matdes.2025.113771.

[77] D. Ma and Y. A. Chang , “Competitive Formation of Ternary Metallic Glasses,” Acta Materialia 54 (2006): 1927–1934, 10.1016/j.actamat.2005.12.015.

[78] S. A. Uporov , V. A. Bykov , E. V. Sterkhov , and I. V. Evdokimov , “Glass Forming Ability in Gd–Co–Al System: Is Vitrification Triggered by Competing Multiple Frustrated Phases?,” Solid State Communications 366‐367 (2023): 115158, 10.1016/j.ssc.2023.115158.

[79] J. J. Han , C. P. Wang , S. Y. Yang , Y. Lu , and X. J. Liu , “Abnormal Orderly Transformation in Supercooled State of an Al‐Based Alloy,” Physical Review Materials 4 (2020): 093608, 10.1103/PhysRevMaterials.4.093608.

[80] J. Z. Zhang and Y. S. Zhao , “Formation of Zirconium Metallic Glass,” Nature 430 (2004): 332–335, 10.1038/nature02715. 15254533

[81] Y. C. Hu , J. T. Zhai , L. H. Liu , et al., “Monatomic Glass Formation through Competing Order Balance,” Nature Communications 16 (2025): 8183, 10.1038/s41467-025-63221-8. PMC12423314 40931093

[82] J. Wang , Z. Q. Cai , H. Kang , et al., “Basic Thermodynamic and Dynamic Characteristics of the Glass Forming Intermetallics,” Materials & Design 238 (2024): 112665, 10.1016/j.matdes.2024.112665.

[83] L. M. Lai , T. H. Liu , X. H. Cai , et al., “High‐temperature Mo‐based Bulk Metallic Glasses,” Scripta Materialia 203 (2021): 114095, 10.1016/j.scriptamat.2021.114095.

