# Supplementary material for: Entropy Decoding the Fundamental Law of Phase Competition in Glass Formation
Source: Adv Sci (Weinh). 2026 Jun 15;13(49):e75936. doi: 10.1002/advs.75936 (PMC13336360; doi:10.1002/advs.75936)
Supplement: Supplementary file 1 [file ADVS-13-e75936-s001.docx]

Supporting Information

**Entropy Decoding the Fundamental Law of Phase Competition in Glass Formation**

*Benke* *Huo, Zhengqing Cai, Bingtao Wang, Zhenqiang Song, Shi-Dong Feng*^*^*,*

*Zijing Li*^*^*, Xingjun Liu, Li-Min Wang*^*^

Supplementary Table S1. Thermodynamic characteristics of intermetallics in Cu-Zr, Ni-Zr, and Cu-Hf systems, Δ*S*_m_ represents the experimental value of melting entropy, and Δ*S*_m_* represents the calculated value of melting entropy using CALPHAD method.

| Compounds | *T*_m_  (K) | Δ*H*_m_  (kJ/mol) | Δ*S*_m_  (J/mol·K) | Δ*S*_m_*  (J/mol·K) |
| --- | --- | --- | --- | --- |
| Ni_7_Zr_2_ | 1712 | 20.36 | 11.89 | 12.04 |
| NiZr_2_ | 1306 | 10.60 | 8.12 | 8.28 |
| Cu_51_Hf_14_ | 1386 | 16.96 | 12.24 | 12.06 |
| Cu_10_Hf_7_ | 1286 | 11.17 | 8.68 | 10.60 |
| CuHf_2_ | 1579 | 27.32 | 17.30 | 17.00 |
| Cu_10_Zr_7_ | 1178 | 7.35 | 6.24 | 8.80 |
| Cu_50_Zr_50_ | 1218 | 7.22 | 5.93 | 6.00 |
| CuZr_2_ | 1283 | 12.4 | 9.67 | 9.00 |

Supplementary Figure S1.  Plots illustrating the relationship between the critical copper-wheel speed (*R*_v-c_) and the parameters: melting entropy (Δ*S*_m_), mixing enthalpy (Δ*H*_mix_), and mismatch entropy ($\text{S}_{\text{σ}}$/*k*_b_).
